# Autistic Traits and Abnormal Sensory Experiences in Adults

**DOI:** 10.1007/s10803-013-2012-7

**Published:** 2013-12-05

**Authors:** Jamie Horder, C. Ellie Wilson, M. Andreina Mendez, Declan G. Murphy

**Affiliations:** 1 Department of Forensic and Neurodevelopmental Sciences, Institute of Psychiatry, King’s College London, De Crespigny Park, PO Box 50, London, SE5 8AF UK; 2 Sackler Centre for Translational Neurodevelopment, Institute of Psychiatry, King’s College London, De Crespigny Park, London, SE5 8AF UK

**Keywords:** Adults, Anxiety, Autism, Comorbidities, Sensory

## Abstract

Sensory processing abnormalities are common in autism spectrum disorders (ASD), and now form part of the Diagnostic and Statistical Manual 5th Edition (DSM-5) diagnostic criteria, but it is unclear whether they characterize the ‘broader phenotype’ of the disorder. We recruited adults (*n* = 772) with and without an ASD and administered the Autism Quotient (AQ) along with the Adult/Adolescent Sensory Profile (AASP), the Cardiff Anomalous Perceptions Scale (CAPS), and the Glasgow Sensory Questionnaire (GSQ), all questionnaire measures of abnormal sensory responsivity. Autism traits were significantly correlated with scores on all three sensory scales (AQ/GSQ *r* = 0.478; AQ/AASP *r* = 0.344; AQ/CAPS *r* = 0.333; all *p* < 0.001). This relationship was linear across the whole range of AQ scores and was true both in those with, and without, an ASD diagnosis. It survived correction for anxiety trait scores, and other potential confounds such as mental illness and migraine.

## Introduction

Autism spectrum disorders (ASDs) are a family of neurodevelopmental syndromes with approximately 1 % prevalence (Brugha et al. 2011; Schieve et al. 2012; Zaroff and Uhm 2011). Currently, ASDs are defined, and diagnosed, on the basis of behaviours, now conceptualized as forming the two domains of: impaired social interaction, language and communication; and restricted interests and repetitive behaviours (American Psychiatric Association 2013; Wing et al. 2011).

However, in addition to these *observable* symptoms, unusual *subjective* sensory and perceptual experiences are increasingly recognized to be key features of ASD. Studies have found high rates of diverse sensory abnormalities in both children and adults diagnosed with an ASD. These include both hypersensitivity and hyposensitivity to various stimuli, as well as strong preferences for or against stimuli that are typically regarded as neutral (Crane et al. 2009; Elwin et al. 2013; Mazurek et al. 2013; Watling et al. 2001).

In a reflection of the increasing research interest in sensory abnormalities, these symptoms are now included in clinical diagnostic algorithms for ASD, namely the Diagnostic and Statistical Manual 5th Edition (DSM-5) and the forthcoming International Classification of Diseases-11 (ICD-11) (Wing et al. 2011) criteria. The assessment of sensory experiences may therefore offer a means of optimizing the diagnosis of ASD, by providing a valuable second source of information alongside the current mainstay of objective behaviour.

However, while the association between ASD and sensory abnormalities is well established, the nature of this relationship is unclear. It is not known whether sensory symptoms are associated with autism spectrum *disorder*, or, more broadly, with ASD *traits*—it now being recognized that ASD symptoms follow a continuous distribution in the population (Baron-Cohen et al. 2001; Le Couteur et al. 1996). One small study (Marche et al. 2012) examined sensory abnormalities in first degree relatives of individuals with ASD, but the authors did not investigate associations with ASD traits. Therefore, from the perspective of assessment, it will be important to ascertain whether sensory abnormalities are indicative of the *presence* of an ASD in a categorical fashion, or alternatively, whether they could serve as dimensional measures of the *severity* of ASD across the whole spectrum.

To date, only one study (Robertson and Simmons 2012) has investigated the relationship between sensory features and broader ASD traits. This reported a significant linear correlation between scores on a novel self-report sensory measure called the Glasgow Sensory Questionnaire (GSQ) and self-reported ASD symptoms as measured using the Autism Quotient (AQ) (Baron-Cohen et al. 2001). This association held in individuals both with and without the disorder, and across the range of AQ scores, not just at the upper end – thus providing preliminary evidence for a dimensional link between sensory abnormalities and ASD traits.

This study was a valuable first step – however, it is unclear whether or not the novel GSQ is comparable to better-validated measures of sensory processing. Furthermore, even if sensory traits are correlated with ASD traits across the whole range, it is unclear whether this indicates a *specific* association between these two constructs. There are other possible explanations - for example, sensory abnormalities might reflect mental health issues commonly comorbid in ASD such as high anxiety (White et al. 2011). Indeed, sensory traits have been shown to be correlated with anxiety severity in children with an ASD (Baker et al. 2008; Ben-Sasson et al. 2008; Lane et al. 2012). Yet no studies have yet examined whether sensory features are correlated with anxiety in adults.

Therefore, in order to replicate the reported relationship between ASD traits and sensory traits across the whole range of severity, and to investigate its specificity, we conducted the largest study to date of autism traits and sensory experiences in a sample of adults both with and without an ASD.

We measured self reported sensory symptoms using both the well-established Adult/Adolescent Sensory Profile (AASP) (Brown and Dunn 2002), and the more recently developed GSQ (Robertson and Simmons 2012). In order to investigate a broad range of unusual experiences we also utilized the Cardiff Anomalous Perceptions Scale (CAPS) (Bell et al. 2006).

Finally, in order to control for a range of potential confounding factors, we recorded the gender and academic speciality of the participants, both of which been linked to ASD traits in student populations (Baron-Cohen et al. 2001). Likewise, we recorded history of mental illness, and migraines, both of which have been associated with self-reported sensory symptoms (Buse et al. 2012; Haigh et al. 2012). We measured anxiety using the trait version of the self-report Spielberger State/Trait Anxiety Inventory (STAI) (Spielberger et al. 1970).

## Methods

### Participants and Demographics

Participants were recruited via an advertisement sent by email to all students and staff of King’s College London (KCL) in London, England. All study questionnaires were administered online, and participants completed them at their own convenience. Participants indicated that they provided informed consent. By taking part in the study, participants were offered the chance to enter a lottery in order to win a monetary prize. No personally identifiable information was collected, except if participants chose to provide it for the purposes of contacting the eventual winner of the prize.

Participants reported their gender, and age in the following bands: 17 or Below, 18-20, 21-25, 26-30, 31-35, 36-40, 41-50, 51-60, 61 or Over. They were also asked to indicate their academic subject (if any), and to answer the following questions:

- Do you have a diagnosis of an autism spectrum disorder (ASD)? ASDs include autism, High Functioning Autism, Asperger’s Syndrome, and Pervasive Developmental Disorder.
- Have any of your immediate family - meaning your parents, brothers, sisters, or children - ever been diagnosed with autism spectrum disorder? (see above).
- Have you ever been diagnosed with any mental health problem, for example: depression, bipolar disorder, or an eating disorder? If so, please give details.
- Do you suffer from regular migraines? If yes, please say roughly how often.

Academic Subject was reported by participants in a text field in which they could freely write a description of their current speciality (if any). Each answer was then coded as being part of 1 of 9 exclusive categories (see Results) by a consensus of two raters, who were blind to all other information about the participants.

### Self-Report Measures

Participants completed the following self-report measures:

The Autism Quotient (AQ) (Baron-Cohen et al. 2001) is a 50-item questionnaire probing core ASD symptoms such as social skill and narrow interests. Each item is scored 0 (non-ASD-like) or 1 (ASD-like) according to whether the response was “Agree” or “Disagree” to each statement. Possible scores: 0 to 50. Example item: *“I find it hard to make new friends.”*

The Glasgow Sensory Questionnaire (Robertson and Simmons 2012) has 42 items probing abnormal sensory behaviours. Each item asks how often the respondent performs a particular behaviour with answers “Never”, “Rarely”, “Sometimes”, “Often” and “Always” being scored 0, 1, 2, 3 and 4 points respectively. Possible scores: 0–168. Example item: *“Do you like to spin yourself round and round?”*

The Adult/Adolescent Sensory Profile (Brown and Dunn 2002) is a 60-item questionnaire probing sensory behaviours. Each item asks how often the respondent performs a particular behaviour with answers “Almost Never”, “Seldom”, “Occasionally”, “Frequently” and “Almost Always” scored 1, 2, 3, 4 and 5 points respectively. Possible scores: 60–300. Example item: *“I stay away from noisy settings.”*

The Cardiff Anomalous Perception Scale (Bell et al. 2006) is a 32-item instrument probing unusual experiences such as heightened sensations or hearing voices. For each item respondents indicate whether it *ever* happens to them, “no” (scored 0) or “yes” (scored 1). Possible scores: 0–32 Note: the CAPS also includes further questions on the frequency, distress and distraction of the experiences (if any) but we did not include these data in the present analysis. Example item: *“Do you ever hear your own thoughts repeated or echoed”?*

The trait version of the Spielberg State/Trait Anxiety Inventory (STAI) (Spielberg et al. 1970) has 20 items dealing with worries and anxiety and asking about how respondents “generally feel”. Answers are “Not at all”, “Somewhat”, “Moderately So” and “Very Much So” and each is scored from 1 (low anxiety) up to 4 (high anxiety), possible scores: 20–80. Example item: *“I feel nervous and restless”.*

Total scores for each questionnaire were calculated using published methods.

The demographic and medical history questions were always presented first; then, the AQ, AASP, CAPS, GSQ, and STAI were presented in random order. The order of the questions within each questionnaire was fixed.

### Statistical Analysis

Only participants who completed all of the measures were included in the analysis (*n* = 772).

#### Primary Analysis

As our objective was to investigate associations between ASD traits, sensory traits, and anxiety, we used Pearson’s correlation coefficient to calculate bivariate correlations between total scores of all five questionnaires. To discover whether any observed correlations were mediated by other variables, we then calculated partial correlations, controlling for whichever trait (autistic, anxiety, sensory) was not involved the bivariate correlation in question.

#### Secondary Analyses

In order to verify that our primary findings were not explained by confounding factors, we recalculated the correlations while controlling for the following variables (coded 0 or 1): gender; any history of mental illness; any history of migraines; any family history of ASD; diagnosis of ASD.

To further investigate whether results differed between those with and without a self reported diagnosis of ASD, we recalculated the correlations separately within the subgroups of individuals, who reported having, and not having, a diagnosis. We also investigated those participants who scored 32 or higher on the AQ (6 %) versus those who scored lower (94 %), as 32 has been proposed as a screening cut-off score on this questionnaire (Baron-Cohen et al. 2001).

As gender is known to be related to autism and autism trait scores (see Introduction), we first compared male to female participants on the outcome measures, using independent-samples *t* tests. We then examined the correlations separately within the male and female participants.

## Results

### Demographics

Of the 772 participants, 555 (72 %) were female and 217 (28 %) were male. For age, the most numerous category was 21–25 years, accounting for 39 % of the participants. 0.5 % were 17 or under, 25 % 18–20, and 18 % 26–30, with 18 % for all ages over 30.

23 (3.0 %) participants self-reported a clinical diagnosis of ASD, and 55 (7.1 %) reported having at least one first-degree relative with diagnosed ASD. 19 % reported having been diagnosed with at least one psychiatric disorder in their lives, and 11 % reported regular migraines.

Coded academic subjects were: medicine and dentistry, 13 %; nursing and midwifery, 11 %; mathematics, physical sciences and engineering, 7 %; biological sciences, 13 %; neuroscience, psychology and mental health, 11 %; humanities, history, human sciences and philosophy, 22 %; arts, 5 %; business, management and law, 6 %; not applicable or not reported, 13 %.

### Primary Results

**Fig. 1:**
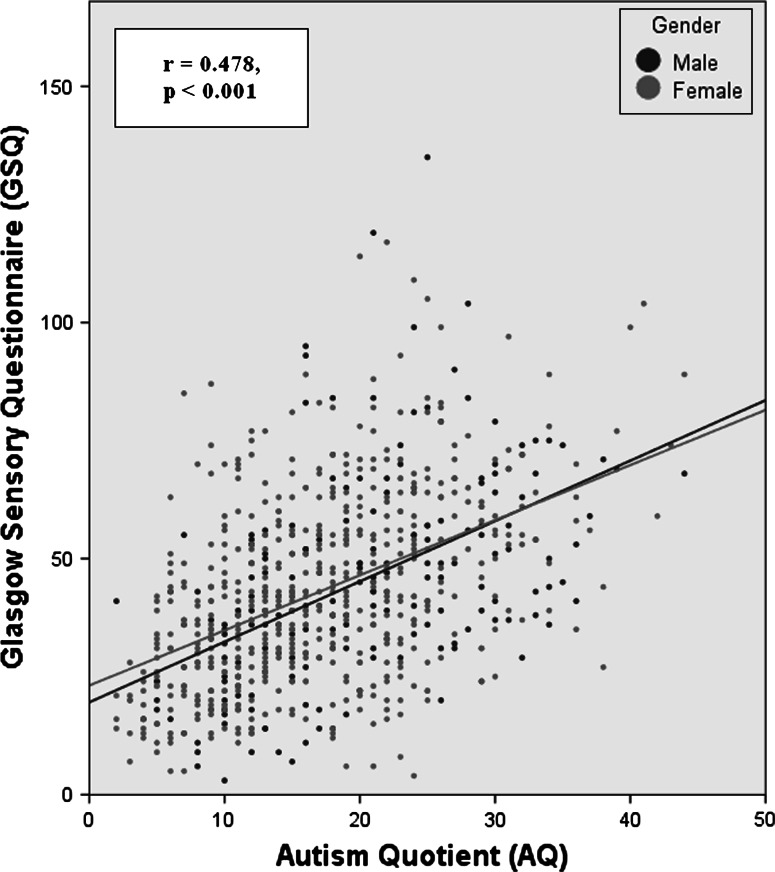
*Scatterplot* showing the relationship between autism trait (AQ) score and sensory (GSQ) score. The two variables have a positive linear association *r* = 0.478, *p* < 0.001 (two-tailed). The relationship is essentially the same in male (*blue dots*) and female (*red dots*) participants showing that the correlation is not the result of confounding by gender

Self-reported abnormal sensory experiences were positively correlated with ASD trait scores, and this was true of all three different sensory questionnaires (AQ vs. GSQ Pearson’s *r* = 0.478; AQ vs. AASP *r* = 0.344; AQ vs. CAPS *r* = 0.333) (see Table 1 and Fig. 1).

**Table 1:** Pearson linear correlation coefficients (*r*) between primary outcome measures

	AQ	GSQ	AASP	CAPS	STAI
AQ					
Pearson *r*	1	0.478*	0.344*	0.333*	0.475*
Sig. (2-tailed)		<0.001	<0.001	<0.001	<0.001
GSQ					
Pearson *r*		1	0.716*	0.547*	0.423*
Sig. (2-tailed)			<0.001	<0.001	<0.001
AASP					
Pearson *r*			1	0.427*	0.408*
Sig. (2-tailed)				<0.001	<0.001
CAPS					
Pearson *r*				1	0.383*
Sig. (2-tailed)					<0.001
STAI					
Pearson *r*					1
Sig. (2-tailed)					

*n* = 772 for all comparisons

Significance values are two-tailed p scores, not corrected for multiple comparisons

*AQ* Autism Quotient, *AASP* Adult/Adolescent Sensory Profile, *GSQ* Glasgow Sensory Questionnaire, *CAPS* Cardiff Anomalous Perceptions Scale, *STAI* Spielberger Trait Anxiety Inventory

* Correlation significant at the 0.05 level (2-tailed)

As expected, all three sensory questionnaires were highly correlated with each other. Anxiety was also correlated with both AQ scores and with sensory behaviours-the first time this has been shown in adults. All of these correlations were statistically significant, *p* < 0.001 two-tailed.

To evaluate whether anxiety explains the relationship between AQ scores and sensory symptoms, we recalculated the correlations between AQ and GSQ, AASP and CAPS using partial correlation controlling for STAI. All three autism-sensory correlations remained significant *p* < 0.001, but were reduced in magnitude (AQ vs. GSQ *r* = 0.346; AQ vs. AASP *r* = 0.184; AQ vs. CAPS *r* = 0.187).

Similarly, we calculated the correlations between anxiety (STAI) and GSQ, AASP, and CAPS, using partial correlation controlling for AQ. All three anxiety-sensory correlations remained significant *p* < 0.001, but were reduced in magnitude (STAI vs. GSQ *r* = 0.254; STAI vs. AASP *r* = 0.184; STAI vs. CAPS *r* = 0.270).

**Fig. 2:**
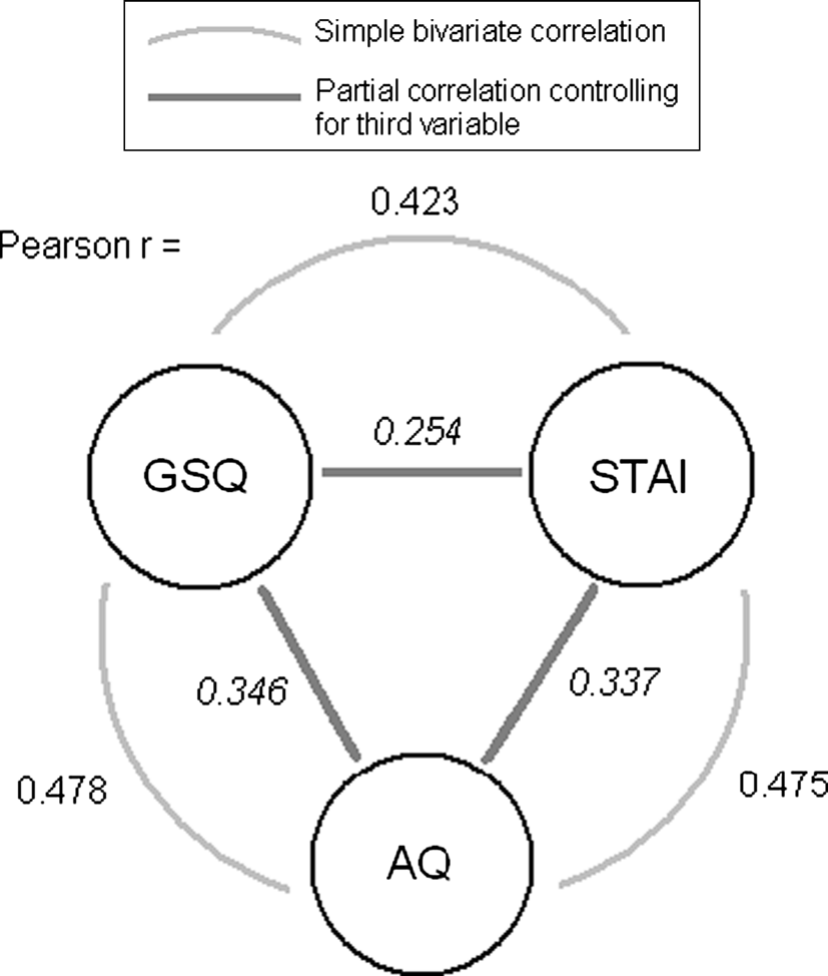
Illustration of the interrelationships between GSQ sensory, AQ ASD trait and STAI anxiety scores. Pearson correlations between each pair of variables are shown, both before and after correction for the third variable

Finally, anxiety remained significantly correlated with autism traits even controlling for sensory scores (AQ-STAI controlling simultaneously for GSQ, AASP and CAPS, *r* = 0.337 *p* < 0.001) (see Fig. 2).

### Secondary Results

#### Controlling for Potential Confounds

We recalculated correlations between the questionnaires controlling simultaneously for: gender; history of mental illness; history of migraines; family history of ASD; and ASD diagnosis. Correlations were essentially unchanged, and all remained significant *p* < 0.001, showing that those potential confounds we measured were not responsible for driving these results.

#### Participants With, and Without, an ASD

In those participants who reported having an ASD, the correlations between the AQ and the three sensory processing measures were significant (*p* < 0.05)—despite the small sample size (*n* = 23)—as was the correlation between anxiety and the AQ. Correlations between the anxiety and the sensory measures GSQ, AASP and CAPS did not reach significance, although the coefficients of correlation were positive (see Table 2).

**Table 2:** Pearson linear correlation coefficients (*r*) between primary outcome measures, in those participants who reported having a diagnosis of ASD

	AQ	GSQ	AASP	CAPS	STAI
AQ					
Pearson *r*	1	0.527*	0.444*	0.652*	0.587*
Sig. (2-tailed)		0.01	0.03	0.001	0.003
GSQ					
Pearson *r*		1	0.640*	0.384	0.352
Sig. (2-tailed)			0.001	0.07	0.1
AASP					
Pearson *r*			1	0.320	0.319
Sig. (2-tailed)				0.136	0.137
CAPS					
Pearson *r*				1	0.378
Sig. (2-tailed)					0.076
STAI					
Pearson *r*					1
Sig. (2-tailed)					

*n* = 23 for all comparisons

*AQ* Autism Quotient, *AASP* Adult/Adolescent Sensory Profile, *GSQ* Glasgow Sensory Questionnaire, *CAPS* Cardiff Anomalous Perceptions Scale, *STAI* Spielberger Trait Anxiety Inventory

* Correlation significant at the 0.05 level (2-tailed)

In those participants without a self-reported ASD, correlations were essentially the same to those seen in the full sample.

We additionally considered participants whose AQ scores were above (*n* = 45) and below (*n* = 727) the widely-used screening cut-off of 32 points (Woodbury-Smith et al. 2005). In participants below the threshold, all of the primary correlations remained significant. Likewise, in those meeting the criteria, the correlations between the AQ and the three sensory questionnaires all remained significant *p* < 0.05 despite the restriction of range and smaller sample size.

We are therefore confident that the association between AQ scores and sensory traits holds across the full range of ASD symptoms.

#### Gender

Compared to females, males scored higher on the AQ (*p* < 0.001, mean 19.6 versus 16.6). We therefore replicate the previously reported gender imbalance in autistic traits scores (Baron-Cohen et al. 2001). However, males did not differ from females on the GSQ (*p* = 0.20), the CAPS (*p* = 0.33), and the STAI (*p* = 0.34). Males scored lower on the AASP (*p* = 0.005, mean 144 vs. 149).

Considering males versus females separately, the correlations between the five questionnaires was substantially identical, and similar to the combined sample (see Fig. 1.)

#### Academic Subject

**Fig. 3:**
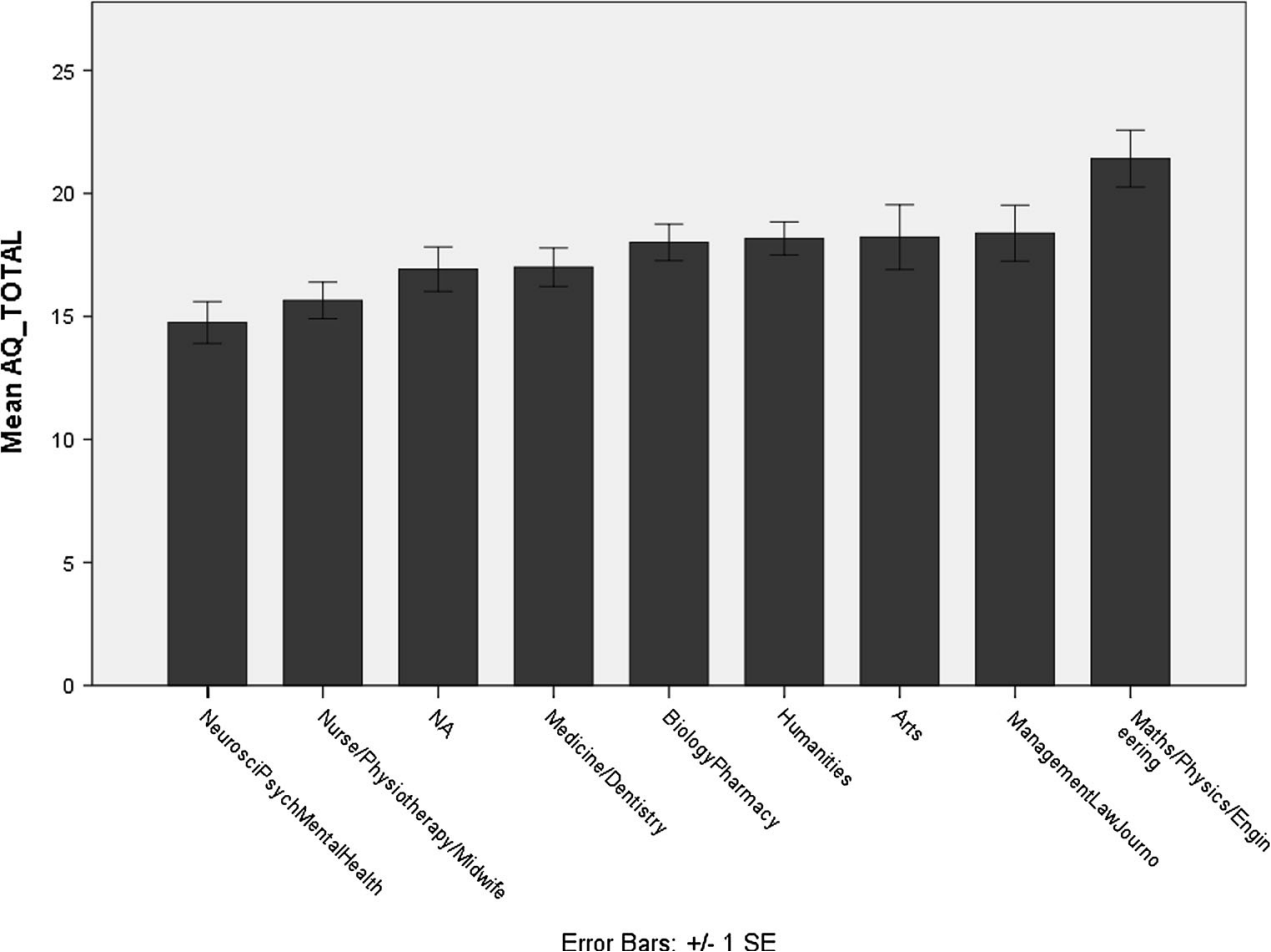
Mean Autism Quotient (AQ) scores by self-reported academic subject

In a general linear model with dependent variable AQ, both of the fixed factors gender (*p* < 0.001) and academic subject (*p* = 0.021) were significant predictors. Inspection of the data show that participants specialized in ‘physical sciences, engineering and mathematics’ scored higher than others, on average. We therefore replicated the reported link between interest in these topics and self-reported ASD traits (Baron-Cohen et al. 2001) (see Fig. 3). However, academic subject was not associated with any sensory scores or with anxiety (all *p* > 0.2).

#### Sensory Traits and Medical History

We examined whether having a history of mental illness, or a history of migraines, were associated with sensory trait scores. Given the finding of an association with AQ scores, we co varied for this factor. A history of mental illness significantly predicted higher scores on all sensory measures, even after controlling for AQ (all *p* < 0.003). Likewise a migraine history predicted higher scores on both the GSQ (*p* = 0.016) and CAPS (*p* = 0.001), but was not a significant predictor of the AASP (*p* = 0.079).

## Discussion

We show that in adults, ASD traits are associated with unusual sensory experiences. These associations were evident within individuals with a self reported ASD diagnosis, those above and below the AQ ‘cut off’ for ASD, and in ‘neurotypical’ controls.

It has previously been reported that ASD traits are associated with sensory scores in the general population (Robertson and Simmons 2012). The present study replicates this finding, and adds to it in several ways: we used three different sensory scales, and confirmed that the association held in each case; and we show that the relationship is not accounted for by the potential confounds of mental illness, migraines, and family history of ASD. Although our sample was predominantly (72 %) female, we found that the correlation between sensory symptoms and ASD traits was very similar in both genders.

Furthermore, we found that ASD traits are correlated with levels of anxiety symptoms, and show using partial correlations that anxiety partially explains the relationship between ASD traits and sensory processing abnormalities—but, even controlling for anxiety, a specific ASD-sensory correlation remained. Likewise, levels of ASD traits mediated the relationship between anxiety levels and sensory processing abnormalities, but only partially. This implies that in ASD, abnormalities with sensory processing and anxiety symptoms are correlated, yet distinct, phenomena.

These results have implications for the proposed integration of sensory processing abnormalities into the diagnosis and assessment of ASD, such as in the DSM-5 criteria (Donnellan et al. 2013; Wing et al. 2011). We confirm the link between sensory experiences and autism across the spectrum, implying that sensory traits could potentially serve as a dimensional measure of the severity of ASD.

However, unusual sensory experiences are not unique to ASD—as we found, trait anxiety, a history of mental illness, and a history of migraines are all also associated with higher sensory scores, even after controlling for ASD traits. Thus, individuals with these conditions may report high levels of unusual sensory experiences, and this could potentially lead to misdiagnosis of ASD, if not controlled for.

In order to achieve the reliable and valid assessment of sensory abnormalities, the choice of measurement instrument will be important. In this study, we used three different questionnaires, the GSQ (Robertson and Simmons 2012), the AASP (Brown and Dunn 2002), and the CAPS (Bell et al. 2006). We found that scores on all three were correlated with ASD traits, but of these the correlation with the GSQ was the strongest at *r* = 0.478 (although not as strong in the original report of this association, in which the correlation coefficient was given as *r* = 0.775 (Robertson and Simmons 2012).)

This implies that the GSQ may be especially appropriate for future research or clinical work on sensory traits in adults with an ASD. Nonetheless, the associations we observed across three instruments (including one, the CAPS, which was designed with psychosis, rather than developmental disorders, in mind) are indicative of the diversity of the sensory experiences linked with the autistic phenotype.

Beyond diagnosis and assessment, these findings could also have implications for treatment interventions. It has been proposed by some—including a number of ASD individuals themselves-that the outer (behavioural symptoms of ASD) represent reactions to inner sensory experiences (Chamak et al. 2008; Elwin et al. 2012; Kientz and Dunn 1997; O’Neill and Jones 1997; Robledo et al. 2012). Prior studies have reported correlations between stereotyped behaviours and severity of sensory features (Boyd et al. 2009). Stereotypies have also been described as ‘sensory modulation behaviours’ (Ben-Sasson et al. 2007) which may serve the function of, for example, avoiding or distracting the individual from aversive experiences in response to their environment.

It is unlikely, in our view, that abnormal sensory experiences underpin *all* ASD symptoms. Nonetheless, it is conceivable that if sensory symptoms could be treated successfully (as in (Schaaf et al. 2013)), some core ASD symptoms—such as stereotypies—would also be reduced (or vice versa). Further research should examine this issue.

The neurobiological underpinning of the sensory abnormalities observed in ASD, and/or their relationship to autistic traits in ‘neurotypicals’, are unknown. A number of possible explanations have been proposed, including greater variability (reduced reliability) in cortical responses to sensory stimuli (Dinstein et al. 2012), atypical lateralization of evoked potentials (Orekhova et al. 2012) and defective inhibition leading to reduced adaptation (Tannan et al. 2008). The latter finding in particular is consistent with the hypothesis of excitatory: inhibitory imbalance in ASD, which in turn are possibly associated with abnormal glutamate or GABA signalling (Coghlan et al. 2012; Horder et al. 2013; Mendez et al. 2012). However, more work is needed to confirm this.

Our investigation has a number of limitations. First, we relied on self-report questionnaires as measures of sensory processing and ASD traits. Although this is a common practice in studies of adults (Chamak et al. 2008; Crane et al. 2009; Robertson and Simmons 2012), it does rely on the participants having insight into their own experiences and being able to verbalize them. In contrast, studies in children have generally used parent-rating instruments, which may provide a more objective measure of sensory-related behaviours-but cannot directly reveal subjective experiences (Brock et al. 2012; Kientz and Dunn 1997; Lane et al. 2012). Therefore, self-report and observer-report are complementary approaches, and future studies should, if possible, utilize both in combination, alongside objective measures of sensory abnormalities e.g. (Blakemore et al. 2006; Panagopoulos et al. 2013).

Secondly, we relied on participants to report their ASD and medical diagnoses. It would be preferable to use medical records or gold-standard diagnostic instruments. It is possible that some participants who reported no diagnosis of, for example, ASD, would receive one were they to be clinically assessed for it. However, we do not consider this to place the fundamental conclusions into question.

A third limitation is that this study was observational, and so we cannot determine the *causal* relationships underlying the correlation between ASD traits and sensory processing. To address this issue, longitudinal studies are required. Nevertheless, adverse reactions to sensory stimuli, if alleviated, could improve an individual’s wellbeing and quality of life.

Finally, our sample was relatively homogenous, in that it was drawn exclusively from staff and students at a single university. In a more heterogeneous population, the associations between autism traits and sensory experiences might be weaker, if other factors are more variable.

In conclusion, ASD traits are associated with sensory processing abnormalities. This is evident in both individuals with an ASD and in ‘neurotypical’ controls. The cognitive/biological underpinnings of this relationship are unknown, and further work is required to determine whether improving sensory processing could reduce the severity of ASD symptoms.
